# Image-guided programming deep brain stimulation improves clinical outcomes in patients with Parkinson’s disease

**DOI:** 10.1038/s41531-024-00639-9

**Published:** 2024-01-27

**Authors:** Viviana Torres, Kirsys Del Giudice, Pedro Roldán, Jordi Rumià, Esteban Muñoz, Ana Cámara, Yaroslau Compta, Almudena Sánchez-Gómez, Francesc Valldeoriola

**Affiliations:** 1 Parkinson’s Disease and Movement Disorders Unit, Neurology Service, Institut de Neurociencies, Hospital Clínic of Barcelona, Barcelona, Catalonia Spain; 2 Neurosurgery Service, Institut de Neurociencies, Hospital Clínic of Barcelona, Barcelona, Catalonia Spain

**Keywords:** Parkinson's disease, Neurodegeneration

## Abstract

Deep brain stimulation (DBS) is an effective treatment for patients with Parkinson’s disease (PD). However, some patients may not respond optimally to clinical programming adjustments. Advances in DBS technology have led to more complex and time-consuming programming. Image-guided programming (IGP) could optimize and improve programming leading to better clinical outcomes in patients for whom DBS programming is not ideal due to sub-optimal response. We conducted a prospective single-center study including 31 PD patients with subthalamic nucleus (STN) DBS and suboptimal responses refractory to clinical programming. Programming settings were adjusted according to the volumetric reconstruction of the stimulation field using commercial postoperative imaging software. Clinical outcomes were assessed at baseline and at 3-month follow-up after IGP, using motor and quality of life (QoL) scales. Additionally, between these two assessment points, follow-up visits for fine-tuning amplitude intensity and medication were conducted at weeks 2, 4, 6, and 9. After IGP, twenty-six patients (83.9%) experienced motor and QoL improvements, with 25.8% feeling much better and 38.7% feeling moderately better according to the patient global impression scale. Five patients (16.1%) had no clinical or QoL changes after IGP. The MDS-UPDRS III motor scale showed a 21.9% improvement and the DBS-IS global score improved by 41.5%. IGP optimizes STN-DBS therapy for PD patients who are experiencing suboptimal clinical outcomes. These findings support using IGP as a standard tool in clinical practice, which could save programming time and improve patients’ QoL.

## Introduction

Deep brain stimulation (DBS) of the subthalamic nucleus (STN) has been shown to improve the quality of life (QoL) of patients with Parkinson’s disease (PD) with motor complications^1^. However, a wide range of factors affect the outcome of patients treated with DBS, including patient selection, target choice, accurate electrode placement, and adequate patient follow-up and optimal programming settings^2^. Inappropriate postoperative management, lead to suboptimal clinical response or adverse effects (AEs)^3–5^, resulting in reduced patient satisfaction and additional follow-up visits^6^.

Conventional clinical programming (CP), regarded as the gold standard for initiating DBS programming, relies on a monopolar assessment based on clinical outcomes. This approach consists of testing each ring contact in a monopolar configuration with the electrode as a negative (cathode) and the implantable pulse generator (IPG) as positive (anode)^5^, evaluating the effect with clinical effect on parkinsonian symptoms and AEs. There are multiple algorithms to develop this strategy but, in general, it is time- and resource-consuming, and is required to be carried out by specially trained clinicians.

Recent advances in DBS technology allow shaping and control of current delivery through different mechanisms including interleaving programming, fractionated current, directional electrodes, anodic stimulation, and reduced pulse width among others. These mechanisms result in an extensive range of parameter combinations of stimulation which increase programming complexity^7^. To address this issue, both freeware (Lead-DBS) (lead-dbs.org; Horn & Kühn. 2017; RRID:SCR_002915), and commercial imaging software tools have been developed, such as Guide™XT (Boston Scientific Corp. Valencia, California, USA) and SURETUNE 4 (Medtronic. Inc., Minneapolis, MN, USA.), which provide precise information on electrode placement based on the patient’s anatomy and simulate the ideal theoretical volume of tissue activated (VTA) in a specific target^8,9^.

Several studies agree that 3D reconstruction and image-guided programming (IGP) strongly correlate with AEs and clinical outcomes^10,11^. This correlation results in non-inferior motor symptom control compared to conventional CP which reduced programming time and improved patient satisfaction^12–16^. For patients with suboptimal outcomes due to the presence of bothersome residual parkinsonian symptoms after multiple programming attempts, the need of a high amount of dopaminergic medication after surgery, or the presence of AEs precluding the possibility of current increases, the use of IGP can be particularly useful.

In this study, we aimed to evaluate the changes in clinical and QoL outcomes using IGP in PD patients with STN-DBS who experienced suboptimal clinical improvement and refractory symptoms with CP.

## Results

### Demographic data

Thirty-one PD patients who underwent STN-DBS treatment were included in the study. Of these patients, 14 (45.2%) were women, and 17 (54.8%) were men, with ages ranging from 41 to 78 years and a mean age of 58.4 ± 8.6 years. The mean disease duration was 13 years ± 6.25, and the mean time from diagnosis to surgery was 11.4 ± 3.84 years. The mean time from surgery until the inclusion in this study was 2.7 years ± 2.8.

### Suboptimal response

Several patients experienced more than one suboptimal response for the control of the symptoms. The most prevalent issue was persisting motor symptoms: residual gait disturbance not attributable to DBS (*n* = 25; 80.6%), followed by residual motor symptoms due to bradykinesia and/or tremors (*n* = 17; 54.8%) and speech disturbance (*n* = 9; 29%).

### Outcomes in quality of life after image-guided stimulation programming

The impact of IGP on QoL outcomes was analysed. On the PDQ-8 scale, patients experienced 38% of improvement (*p* = 0.001) after IGP, with scores changing from 36.2 ± 16.0 at baseline to 21.7 ± 13.8 after programming. The EQ-VAS scale showed an improvement of 31.6% (*p* = 0.001), with the initial mean score of 4.35 increasing to 6.77 after IGP, indicating an average improvement of two points towards better health status on the analog scale.

At baseline, the PGI-S severity of the disease score ranged between 4.0 (Moderately ill) and 5.0 (Severely ill), with a mean of 4.3 ± 0.4. The PGI-improvement score had an average of 2.4 ± 1.3, with 25.8% (*n* = 8) reporting much better, 38.7% (*n* = 12) reporting moderately better, 12.9% (*n* = 4) reporting slightly better, 6.5% (*n* = 2) reporting no change, and 16.1% (*n* = 5) reporting moderately worse.

Table 1 summarizes the scores of the scales evaluated and the percentage of improvement achieved in each variable.

**Table 1:** Clinical data of the suboptimal DBS PD patients.

ID	LEDD pre(mg)	LEDD pos(mg)	DBS- IS Pre	DBS- IS Pos	UPDRS III pre	UPDRS III pos	PDQ-8 pre	PDQ-8 pos	EQ-VAS pre	EQ-VAS pos	PGI-S Pre	PGI-I Pos	VTA Left pre	VTA Left pos	VTA Right pre	VTA Right pos
1	300	0	28	13	22	19	28	13	4	7	4	2	0.15	0.07	0.09	0.09
2	0	0	13	3	16	11	22	0	4	8	4	1	0.1	0.07	0.19	0.13
3	750	750	32	8	16	6	31	0	3	8	5	1	0.16	0.17	0.12	0.14
4	300	300	20	22	20	22	31	31	6	8	4	5	0.15	0.15	0.06	0.14
5	350	350	11	11	26	26	16	16	4	4	4	4	0.05	0.06	0.17	0.05
6	0	0	40	13	10	10	19	6	6	7	4	2	0.09	0.07	0.09	0.07
7	150	80	27	14	20	12	59	19	4	7	4	3	0.12	0.12	0.07	0.08
8	176	176	27	19	20	11	56	22	4	6	4	3	0.05	0.08	0.05	0.05
9	0	0	18	9	18	13	59	9	5	7	4	2	0.25	0.28	0.12	0.12
10	300	300	49	38	28	20	59	56	2	5	5	3	0.07	0.1	0.13	0.14
11	400	400	20	13	23	23	56	47	4	4	4	4	0.06	0.06	0.12	0.12
12	400	400	23	10	18	10	38	19	4	7	4	2	0.09	0.09	0.11	0.12
13	600	600	28	19	32	26	34	25	4	7	4	2	0.09	0.09	0.09	0.09
14	650	500	32	17	18	12	59	38	4	7	5	2	0.06	0.14	0.11	0.15
15	52	52	16	10	15	6	16	9	5	9	4	1	0.15	0.15	0.17	0.17
16	700	700	45	22	16	17	59	31	5	7	5	2	0.17	0.17	0.15	0.13
17	500	400	26	16	15	13	31	25	5	6	5	3	0.13	0.13	0.09	0.08
18	1773	1773	28	18	28	23	38	31	6	8	4	2	0.12	0.1	0.12	0.09
19	1000	400	28	10	4	3	19	13	5	8	4	2	0.06	0.08	0.04	0.07
20	800	600	23	23	16	16	22	22	5	5	5	5	0.1	0.11	0.08	0.09
21	0	0	39	8	16	14	25	19	6	9	5	1	0.12	0.14	0.08	0.09
22	399	399	29	16	49	28	50	41	4	7	5	2	0.14	0.18	0.18	0.21
23	1200	0	24	9	23	16	25	13	5	7	4	1	0.19	0.18	0.19	0.16
24	500	500	22	21	15	22	25	28	5	4	4	5	0.05	0.05	0.05	0.05
25	1000	1000	18	9	15	6	44	13	2	8	4	1	0.11	0.11	0.07	0.08
26	500	225	24	12	28	16	59	25	1	7	5	2	0.08	0.09	0.11	0.11
27	870	870	19	19	16	17	9	9	5	5	4	5	0.07	0.09	0.09	0.03
28	1800	1800	23	22	23	25	38	44	6	4	4	5	0.02	0.06	0.03	0.03
29	150	100	23	11	24	12	44	19	4	8	4	1	0.22	0.14	0.06	0.15
30	800	800	28	17	29	26	34	28	4	8	5	2	0.12	0.11	0.1	0.12
31	150	150	17	2	21	11	16	3	4	8	4	1	0.13	0.1	0.12	0.12
Mean	534	439	25.8	14.6	20.6	15.8	36.2	21.7	4.3	6.7	4.3	2.4	0.1119	0.1142	0.1048	0.1055
SD	467	457	8.6	7.04	7.9	6.8	16.0	13.8	1.1	1.4	0.4	1.39	0.05	0.04	0.043	0.04
Overall % of improvement	LEDD: 14.8%		DBS - IS: 41.5%		UPDRS III: 21.9%		PDQ 8: 38%		EQ 5D: 31.6%							

Including scores of evaluated variables and the global percentage of improvement.

*EQ-VAS* EuroQol visual analog scale, *PDQ-8* The 8-item version of the Parkinson’s Disease Questionnaire, *DBS-IS* Deep Brain Stimulation Impairment Scale d, *PGI-I* The Patient Global Impression scale improvement. *LEDD* levodopa equivalent daily dose; UPDRS III. Movement Disorders Society-Unified Parkinson Disease rating scale. *VTA* Volume Tissue activated.

### Outcomes in motor symptoms and levodopa equivalent daily dose

The DBS-IS global score was 25.8 ± 8 at baseline, significantly decreasing to 14.6 ± 7 after programming, resulting in an average improvement of 11 points (41.5%) (*p* = 0.001). (Table 1) The MDS-UPDRS III score decreased significantly by 5 points (21.9%) (*p* = 0.001), from an initial score of 20.6 ± 7.9 to 15.8 ± 6.8 after IGP (Table 1).

We also observed significant changes in levodopa equivalent daily dose (LEDD) after stimulation adjustments, with the mean pre-LEDD of 534.5 mg decreasing to 439.5 mg post-programming (*p* = 0.008). Of this group, 6.5% (*n* = 2) discontinued medication completely, 25.8% (*n* = 8) dropped at least half of the dose, and the remaining 67% (*n* = 21) continued with the same previous amount (Table 1).

### Programming adjustments and VTA

All 31 patients underwent programming adjustments; in 10 patients these adjustments involved changes in only one of the two implanted electrodes, right or left. Most changes consisted of current directionality, with 37% (*n* = 23) of electrodes receiving changes in vertical and horizontal directions. Contact changes were made in 40% (*n* = 25) of the electrodes, amplitude increases for 7% (*n* = 4) and no adjustments were made for 16% (*n* = 10) of the electrodes (Table 2).

**Table 2:** Programming parameters before and after IGP.

ID	Initial stimulation parametrers					Optimal stimulation parametrers					Programming change
	Side	Contact	A(mA)	FC (Hz)	Pw (mcs)	Side	Contact	A(mA)	FC (Hz)	Pw (mcs)	
1	L	Ventral supraventral	4.6	130	60	L	Subdorsal	2.4	130	60	Directional/Steering
	R	Subdorsal	2.9	130	60	R	Supraventral lateralized	3.5	130	50	Contact change
2	L	Ventral	3	130	60	L	Supraventral, subdorsal	2.6	130	60	Contact change
	R	Supraventral, subdorsal	5	130	60	R	Supraventral, subdorsal (lateralized)	3.5	130	60	Directional/Steering
3	L	Ventral,supraventral (medial)	6	130	40	L	Steering vertical 10% ventral and 90 supraventral (antero-medial)	6.5	130	30	Directional/Steering
	R	Ventral	5	130	40	R	supraventral(90%) and subdorsal(10%) (Medial)	5.5	130	40	Directional/Steering
4	L	Supraventral	4.2	130	60	L	Supraventral, subdorsal	4.2	130	60	Directional/Steering
	R	Supraventral lateral	3.8	130	60	R	Supraventral,subdorsal (lateralized)	3.8	130	60	Directional/Steering
5	L	Ventral	2	130	60	L	Ventral	2.1	130	30	None
	R	Subdorsal	4.8	130	60	R	Ventral	1.9	130	40	Directional/Steering
6	L	Supraventral	3	130	60	L	Supraventral, subdorsal	2.4	130	60	Directional/Steering
	R	Supraventral	3	130	60	R	Supraventral, subdorsal (lateralized)	2.5	130	60	Directional/Steering
7	L	Supraventral, medial	3.5	130	60	L	Subdorsal	3.6	130	60	Directional/Steering
	R	Supraventral	2.7	130	60	R	Supraventral	2.8	130	60	Directional/Steering
8	L	Supraventral	2.1	130	60	L	Supraventral	2.1	130	60	None
	R	Supraventral	2	130	60	R	Ventral, supraventral	1.9	130	60	Directional/Steering
9	L	Subdorsal	6.3	180	60	L	Subdorsal (70%)Dorsal (30%)	6.8	180	60	Directional/Steering
	R	Subdorsal	3.2	180	60	R	Subdorsal	3.7	180	60	None
10	L	Subdorsal	2.6	200	60	L	Subdorsal	3.2	200	60	Voltage change
	R	Subdorsal	3.8	200	60	R	Dorsal	4	200	60	Contact change
11	L	Subdorsal	2.4	180	60	L	Supraventral	2.4	180	60	Contact change
	R	Supraventral	4	180	60	R	Supraventral	4.2	180	60	None
12	L	Supraventral	3.0	120	60	L	Supraventral	3.0	120	60	Directional/Steering
	R	Supraventral	3.4	120	60	R	Ventral	3.4	120	60	Contact change
13	L	Dorsal	3.0	130	60	L	Subdorsal	3.0	130	60	Directional/Steering
	R	Subdorsal	2.7	130	60	R	Supraventral	3.0	130	60	Contact change
14	L	Supraventral	2.4	130	60	L	Subdorsal	5.5	130	50	Directional/Steering
	R	Supraventral	3.4	130	60	R	Supraventral	7	130	50	Directional/Steering
15	L	Ventral	4.3	130	60	L	Supraventral	4.3	130	60	Contact change
	R	Ventral	4.7	130	60	R	Ventral	4.7	130	60	Contact change
16	L	Subdorsal	4.6	120	60	L	Subdorsal	4.6	120	60	Directional/Steering
	R	Subdorsal	4,3	120	60	R	Supraventral	3.8	120	60	Directional/Steering
17	L	Supraventral	3.8	130	60	L	Supraventral	3.8	130	60	Directional/Steering
	R	Subdorsal	3.0	130	60	R	Supraventral	2.8	130	60	Contact change
18	L	Ventral	3.5	60	60	L	Ventral	3.0	120	60	Voltage change
	R	Ventral	3.5	60	60	R	Supraventral	3.0	120	60	Contact change
19	L	Subdorsal	2.3	130	60	L	Supraventral	2.8	130	60	Contact change
	R	Ventral	1.7	130	60	R	Ventral	2.5	130	60	Voltage change
20	L	Supraventral	3.2	130	60	L	Subdorsal	3.4	130	60	Contact change
	R	Supraventral	2.8	130	60	R	Supraventral	3.0	130	60	None
21	L	Supraventral	3.7	130	60	L	Supraventral, subdorsal	4.0	130	60	Directional/Steering
	R	Supraventral	2.9	130	60	R	Supraventral	3.1	130	60	None
22	L	Supraventral	4.8	185	60	L	Ventral	6.3	185	70	Contact change
	R	Supraventral	4.8	185	60	R	Ventral	5.3	185	60	Contact change
23	L	Dorsal, Subdorsal	5	128	60	L	Subdorsal	4.0	128	60	Contact change
	R	Dorsal, Subdorsal	5	128	60	R	Dorsal	4.5	128	60	Voltage change
24	L	Ventral	2	130	60	L	Ventral	2	130	60	Directional/Steering
	R	Supraventral	1.5	130	60	R	Supraventral(Lateralized)	2	130	60	Contact change
25	L	Supraventral	3.2	130	60	L	Ventral	3.4	130	60	None
	R	Supraventral	2.6	130	60	R	Supraventral	2.9	130	60	Contact change
26	L	Supraventral	2.9	130	60	L	Supraventral, subdorsal	2.9	130	60	Directional/Steering
	R	Ventral	3.5	130	90	R	Supraventral, subdorsal	3.4	130	60	Contact change
27	L	Subdorsal	5	126	60	L	Subdorsal	5	126	60	None
	R	Subdorsal	4.5	126	60	R	Supraventral	4.5	126	60	Contact change
28	L	Dorsal	1.9	130	60	L	Supraventral, subdorsal (lateralized)	2	130	60	Contact change
	R	Supraventral, subdorsal (lateralized)	3.2	130	40	R	Supraventral, subdorsal (lateralized)	3.2	130	60	None
29	L	Bipolar supraventral subdorsal (-)	3	159	90	L	Ventral (10%)supraventral (80%)	3	130	60	Contact change
	R	Supraventral subdorsal	4	159	90	R	Ventral (10%) supraventral (80%)	2.5	130	60	Contact change
30	L	Dorsal	3.6	128	60	L	Supraventral	3.6	128	60	Contact change
	R	Dorsal	3.3	128	60	R	Supraventral	3.3	128	60	Contact change
31	L	Supraventral	3.7	130	60	L	Supraventral	3.7	130	60	Contact change
	R	Supraventral	2	130	60	R	Dorsal	2	130	60	None

The table displays the contact information and programming parameters such as Amplitude (A) measured in mA, Pulse Width (PW) measured in microseconds (mcs), and Frequency (FC) measured in Hertz (Hz). Moreover, the table provides a clear indication of the programming modifications implemented, along with the identification of the contacts based on anatomical classification, including ventral, supraventral, subdorsal, and dorsal.

During the follow-up visits, it was found necessary to make slight increases in the amplitude of 77% (*n* = 48) of the electrodes in week 2, 60% (*n* = 37) of the electrodes in week 4, and 47% (*n* = 29) of the electrodes in week 6. These adjustments involved raising the current by no more than 0.3 mA. Importantly, there were no modifications made to pulse width, frequency, or contacts during these visits. No adjustments were made in visits week 9 and 12.

After adjustments, the mean VTA for the left STN was 114.2 mm^3^, and for the right STN was 105.5 mm^3^, compared to pre-adjustment values of 111.9 mm^3^ and 104.8 mm^3^, respectively. However, no statistically significant differences were found for the left and the right VTA (*p* = 0.384 and 0.688, respectively) (Table 1).

The stimulation parameters and the programming changes made before and after IGP are listed in Table 2.

**Fig. 1:**
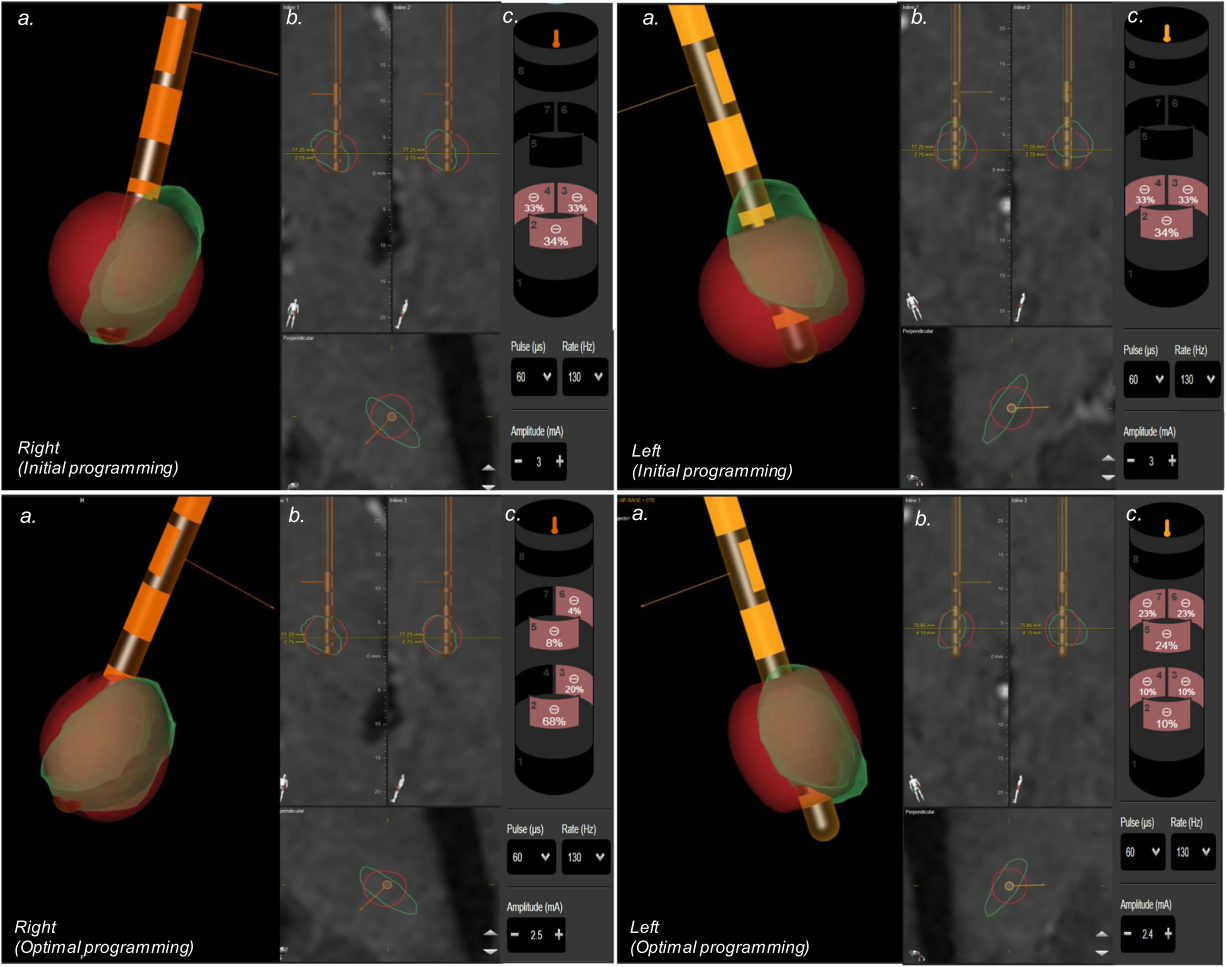
Simulation of stimulation patient 5. Residual symptoms after surgery primarily include freezing of gait. **a** 3D reconstruction of the STN (Green), electrode position (Orange), and VTA model (red). **b** Inline, perpendicular and axial view. **c** Programming settings. The top panel illustrates the initial stimulation. The left electrode is located medially to the STN, with circular stimulation in supraventral contact, while the VTA extends beyond the STN boundaries medially. The right electrode is in the appropriate position. With supraventral contact stimulation, the VTA is situated ventrally in relation to the NST. The bottom panel demonstrates image-guided programming to cover the NST region. In the left electrode, vertical current direction is applied in supraventral and sudorsal contact, with lateralization of the current. In the right electrode, vertical steering is applied in supraventral and subdorsal contact.

**Fig. 2:**
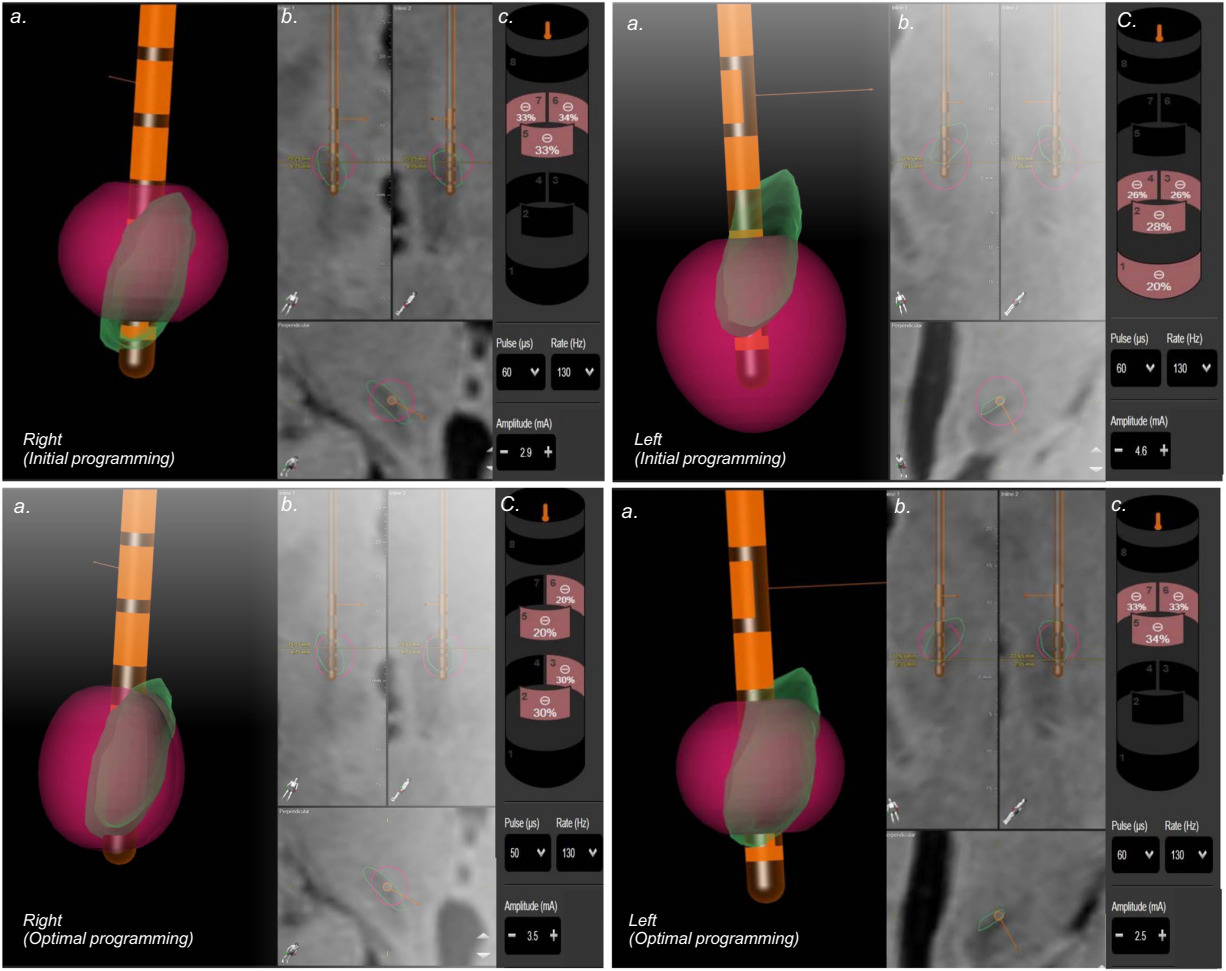
Stimulation simulation patient 1 residual symptoms following surgery, primarily related to motor symptoms of freezing of gait. **a** 3D reconstruction of the STN (Green), electrode position (Orange), and VTA model (red). **b** Inline, perpendicular and axial view. **c** Programming settings. In the upper panel, the image illustrates the initial stimulation programming. The right electrode is positioned slightly medially to the subthalamic nucleus (STN) with circular stimulation in a subdorsal contact. In the axial plane, the VTA overlaps the dorsal portion of the STN. On the left side, the electrode is correctly positioned, and the stimulation is located ventrally relative to the nucleus. The lower panel demonstrates the image-guided programming aimed at covering the dorsolateral STN region. At the right electrode, the vertical direction is applied at the supraventral and ventral contacts. The left contact underwent a contact change (subdorsal), primarily targeting the dorsolateral portion of the nucleus.

Representative cases of reconstructions are illustrated in Figs. 1, 2, showcasing the simulation of stimulation characteristics and initial VTA parameters leading to suboptimal outcomes, as well as the parameters guiding VTA at the target.

## Discussion

In our cohort IGP has proved useful as a tool to improve QoL and motor outcomes in patients with PD and STN-DBS who had a suboptimal response in specific clinical aspects. Clinical benefits could be observed in some patients after several years of DBS surgery and multiple attempts with CP. Improvements were present acutely following optimization and were maintained at the 3-month follow-up compared to baseline assessments.

Defining what constitutes a suboptimal outcome is complex. Tagliali et al. highlight how this complexity stems from the multifaceted nature of PD. It extends beyond motor impairments, encompassing non-motor aspects such as depression, sleep disturbances, dysarthria, autonomic dysfunction, and sensory symptoms^17^. These dimensions, often not the primary targets of DBS further complicate assessing therapeutic success.

Optimal clinical outcomes with DBS strongly depend on the location of the electrodes^18^ and the programming of stimulation parameters used to generate an electric field in the targeted area. Despite proper patient selection and correct implantation, inefficient programming can significantly hinder success accounting for up to 37–52%^19,20^ of DBS failures.

Multiple factors can contribute to ineffective programming, such as limited time or resources at the treatment center, complex cases, hardware failures, and a lack of experience in centers with low annual implantation rates. To achieve the best results, it is crucial to optimize stimulation parameters and precisely shape the electric field within the intended target area, while carefully avoiding undesired regions that could trigger unintended effects. Consequently, numerous technological advancements have emerged in DBS, aiming to direct the electrical current to an ideal “sweet spot”^21,22^. However, these advancements have also introduced additional challenges in programming the electrodes.

Efforts have been made to determine the optimal localization of the stimulation^18,23,24^. When the electrical stimulation is within the dorsolateral part of the STN, contralateral PD symptoms can improve by up to 71.5%, significantly reducing levodopa dosage by up to 77%^25^. However, there is still considerable variation in target localization among DBS specialists, and a consensus on the optimal anatomical target has yet to be established^26^. The results of our work are consistent with these observations, as in the study also targeted the dorsolateral portion of the STN resulting in a 22% improvement in the UPDRS III motor scale.

The advancement of imaging technologies that leverage patient-specific anatomy has facilitated the precise identification of electrodes and electric fields within the nucleus. In this study, we utilized the commercial software GUIDE™ XT, which offers an intuitive visualization of electrode positioning within the STN. It also establishes its spatial relationship with adjacent structures and incorporates the simulation of the VTA, all based on stimulation parameters.

Previous studies on GUIDE™ XT have shown good concordance with the automatic segmentation performed by the system with the anatomical boundaries defined by microelectrode recording (MER) data, supporting the accuracy of the obtained images^27^. In the clinical field, GUIDE™ XT has proven to be a valuable tool for achieving clinical improvement compared to the traditional trial and error approach but with shorter and more efficient programming sessions^14^.

Numerous studies have consistently demonstrated that the use IGP for treating motor symptoms is comparable to traditional CP in terms of symptom control^12,14,15,28^. IGP has also been shown to shorten total programming time by requiring fewer programming sessions and less discomfort for patients^28–30^. To our knowledge, the potential of IGP used to optimize clinical outcomes in PD patients with suboptimal symptom control has not been well studied before.

In this work, the most frequent programming changes were directional programming (37%) and contact change (40%). Precise directional programming is an extremely laborious and practically infinite process with CP due to the multiple possibilities offered by the system. This study evidenced that the clinical improvements achieved with IGP was maintained after 3 months, with minor changes in mA. These results reasonably rule out confounding the improvement with a possible placebo effect.

As a result, this study showed significant improvements in disease-specific QoL scales and remarkable improvements in the DBS-IS scale, especially in speech and gait symptoms, which were commonly reported as suboptimal. The study found statistically significant differences on the PDQ-8 and EQ-5D scales; and 33% of patients were able to reduce their LEDD by discontinuing or reducing their medication by at least half.

In this study, five patients lacked response to IGP strategies. This could be a result of several factors, including the duration of the disease and the duration of DBS therapy which may influence the effectiveness of the treatment. It is also possible that patients’ expectations for the settings were too high, leading to lower scores on QoL scales. Additionally, the “sweet spot” location for suboptimal symptoms that did not improve may differ from the area targeted by the programming.

The small sample and the single-center nature are an acknowledged limitation of the study. This circumstance introduces potential variability attributed to factors such as the composition of the surgical team, the employed strategies for stimulation programming, and the collective years of experience for the individuals involved. An additional limitation is the electrode placement procedure, which relied on general anesthesia with image guidance (IMG) and lacked microelectrode (MER) recordings and intraoperative clinical assessment. However, there are multiple studies that consistently demonstrate that IMG-guided procedures produce similar clinical results on the precision and accuracy of electrode placement in terms of safety, precision, and efficacy to MER-guided approaches^31,32^. Another limitation of this study is that motor fluctuations were not evaluated, since this was not our primary objective, we targeted patients experiencing dissatisfaction in QoL with persisting motor symptoms and/or inadequate dopaminergic response, and therefore the selected patients did not experience clear motor fluctuations.

In conclusion, our study provides evidence that IGP can enhance cases previously considered suboptimal responders under CP alone. IGP resulted in a valuable tool to improve the clinical outcomes and patient’s QoL in our study. To validate the study findings, further research is essential. Future randomized clinical trials, involving larger samples across multiple centers, could confirm IGP’s efficacy in treating specific symptoms compared to CP, both in outcomes and efficiency. This approach could pave the way for refined personalized treatment, significantly elevating patient care standards.

## Methods

Between December 2021 and December 2022, we conducted a prospective study at the Hospital Clinic of Barcelona with patients with PD who had undergone bilateral STN-DBS implanted with a Boston Scientific DBS system. Participants enrolled in this study met tailored inclusion criteria designed to identify suboptimal outcomes after DBS. These criteria consider the complex nature of PD, extending beyond traditional motor assessments.

The inclusion criteria were: **1. Suboptimal clinical outcomes** after adequate and specialized clinical follow-up, involving at least three attempts with CP during follow-up visits. These involved patients who presented at least one of the following situations: **1.1 Persisting Motor Symptoms:** Participants exhibiting persistent motor symptoms after DBS, such as gait disturbances, speech impairments, bradykinesia, or tremors. This criterion is predicated on less than 30% improvement in the MDS-UPDRS III scale compared to their condition in the stim OFF state. **1.2 Inadequate Dopaminergic Medication Response:** Patients needing substantial post-DBS dopaminergic doses, with less than 50% reductions from pre-surgery dosage. This recognizes cases where expected medication reduction hasn’t occurred. **1.3 Subjective Patient Dissatisfaction and Quality of Life (QOL):** This includes patients perceiving unsatisfactory outcomes despite meeting objective success standards. It evaluates QOL impact and considers patient-reported dissatisfaction from unaddressed non-motor symptoms, reflecting personalized suboptimal outcome interpretation. In alignment with quality of life assessment scales, we considered the following thresholds: EQ5-VAS Score: participants with a score of less than 7 on a scale ranging from 0 to 10 and/or PDQ8 Score: participants with a score exceeding 16 on a scale of 0 to 100. **2. Correct placement of the electrodes**, known cases of malpositioning of at least one of the implanted electrodes were excluded.

The study was conducted following the Declaration of Helsinki and was approved by the local ethics committee at the Hospital Clinic of Barcelona. All patients gave informed written consent before they participated in the study.

### Surgical procedure

As part of the pre-surgical protocol, all patients underwent a 3-Tesla magnetic resonance imaging (MRI) under sedation, with targeting performed using in parallel both, the StealthStation S8 (Medtronic Inc., Minneapolis, MN, USA) and the BrainLab Elements® software (BrainLab AG, Much, Germany). MRI-direct visual anatomical targeting of the Subthalamic Nucleus (STN) was used. The stereotactic frame (Leksell-G; Elekta AB, Stockholm, Sweden) was fixed, and stereotactic coordinates were obtained from stereotactic computed tomography (CT) and image datasets coregistration. Electrode placement for PD in the STN was performed under general anesthesia, using a direct electrode placement method without microelectrode recordings. Intraoperative verification of electrode placement was done using the O-arm 3D fluoroscopic imaging system (Medtronic Inc., Minneapolis, MN, USA). After electrode placement, extension wires and an implantable pulse generator were implanted, and the electrode position was verified using postoperative 3D computed tomography co-registered utilizing preoperative magnetic resonance planning images.

### Imagine software

3D image reconstruction was performed using the commercially available software GUIDE™XT from Boston Scientific Corp., Valencia, California, USA. The sequences used include 3D sagittal FLAIR-T2 and 3D T1 with gadolinium and postoperative 3D helical brain CT. These images are automatically merged into the software using a coregistration algorithm, and anatomical mapping of the STN was performed using anterior-posterior commissure (ACPC) positioning, which was verified by the neurosurgical team.

The software assessed the electrode position and orientation based on the subsequent CT scan artifact. Finally, the simulation tool was used to create potentially effective stimulation settings (contacts, direction, and the parameter configuration: current amplitude, pulse width and frequency) that resulted in the volume of the electrostatic field (VEsF) representing the volume of tissue activated (VTA).

The VTA model is comprised of two primary components. The first is an electrical model, where a three-dimensional finite element mesh is constructed to represent neural tissue^33^. The second, is a detailed axon model, governed by differential equations, simulates ion flow in CNS axons. These equations detail the flow of ions through ion channels found in the central nervous system’s axons^34^. Integrating extracellular potentials from the electric field model into this axon framework facilitates a grounded assessment of the likelihood of an action based on the set parameters. The resulting locations where the model predicts potential action potential events are utilized to create the practical and clinically relevant 3D contour^35^.

The calculations of object volumes (mm^3^), as well as the generation of intersection and union objects, were executed through the application of the object manipulation component developed by Brainlab in Munich, Germany.

Two VTA reconstructions were performed to evaluate the effects of DBS on the patient’s symptoms. The first reconstruction occurred during the baseline phase when the patient was experiencing suboptimal symptoms in outpatient follow-ups. The initial reconstruction used the same active stimulation parameters when the patient met the inclusion criteria and was enrolled in the study.

The subsequent reconstruction aimed to determine the optimal configuration for the patient’s condition. The study´s focus was specifically on the dorsolateral part of the subthalamic nucleus (STN), a region previously associated with the most favorable clinical motor outcomes^20^. In this adapted simulation, the study maintained a similar VTA compared to the baseline stimulation. However, the main adjustment consisted of redirecting the stimulation current toward the dorsolateral portion of the STN, thus improving the accuracy of the stimulation direction to the theoretical “sweet location”.

### Basal stimulation configuration parameters

The initial patient parameters were derived from the monopolar review. This is a process aimed to identify and select the optimal contact point while screening for acute adverse effects. Initially circular stimulation was assessed across all four levels, followed by an individual evaluation of directional contacts to ensure precise targeting of therapeutic effects.

The stimulation amplitude was systematically raised in 0.5 mA increments, culminating at 4 mA as the upper limit or until AEs appeared. Stimulation was configured on the contact demonstrating the broadest therapeutic window at lower intensities while necessitating higher intensities for the emergence of adverse effects. The programmed stimulation represented the minimal level required to effectively manage Parkinsonian symptoms, with vigilant monitoring of therapeutic impacts and potential side effects maintained during this phase.

Furthermore, as part of the clinical management programming routine, follow-up visits were conducted for adjustments. Despite numerous revisions, the patients experiencing suboptimal outcomes were identified and selected for IGP protocol.

### Image-guided programming protocol

*Simulation of stimulation (VTA-based)*. The GUIDE XT™-derived programming was performed by the DBS expert neurologists blinded to the CP and its resulting outcomes. The blinded procedures were conducted by the following individuals: KDG (clinical assessments), VT (programming), AS (GUIDE XT) and FV (follow-up visits). Patients were not blinded to the intervention, they were informed of the possible programming adjustments that would be made, but they were not informed of the specific changes made or lack thereof.

Utilizing the GUIDE XT™ image reconstruction, the research visually identified the dorsolateral part of the STN, referred to as the optimal location, which served as the basis for creating a VTA. By adjusting the active contacts and directionality, the dorsolateral section of the STN was precisely targeted while preserving neighboring structures. The VTA simulation of stimulation was conducted using programming parameters set at an amplitude of 1 mA below baseline, a pulse width of 60 μs, and a frequency of 130 Hz.

#### Image-based programming

Initially, the patients were clinically evaluated by neurologists with expertise in movement disorders with basal parameters. Reprogramming was performed based on the stimulation parameters that were considered optimal, derived from the 3D reconstruction.

The GUIDE XT™-derived program was sequentially initiated at 1 mA below baseline, incrementally raising the amplitude by 0.2 mA steps until reaching the initially employed threshold voltage or below it. The decision on the total current was guided by its clinical effectiveness. The standard frequency and pulse width settings (130 Hz, 60 μs) remained unaltered initially. After switching to IGP, patients were observed for 2 hours to check for any immediate adverse effects. Adjustments to pulse width were allowed if adverse events occurred, but changes to stimulation direction or active contacts were not permitted during the study.

Throughout this process, meticulous monitoring of the clinical response and occurrence of acute AEs were tested and documented. Acute AE was defined as those that appeared within 48 h after the GUIDE XT™-derived program, and chronic AE as those that appeared in the follow-up at three months. Immediate improvement was evaluated. In case of worsening symptoms or adverse effects the previous program was reinstated. Additionally, we investigated the potential synergistic effects of levodopa treatment.

### Clinical assessment

Demographic and clinical data, including age, sex, disease duration, type of PD symptoms (akinesia, rigidity, tremor, and gait disorder), pre-surgery dopaminergic medication, and age at the time of surgery were obtained. Postoperative clinical data was prospectively collected, including the time from surgery to inclusion in this study, stimulation parameters, current dopaminergic medication, and the reason for defining a suboptimal outcome.

All patients underwent baseline and three-month follow-up evaluations after IGPg using specific scales administered by a movement disorders specialist. The scales used were: (1) Movement Disorders Society-Unified Parkinson Disease rating scale subitems of the UPDRS Part III (2). Deep Brain Stimulation Impairment Scale (DBS-IS). This scale has been developed and validated to assess motor and non-motor impairment on different subitems such as postural instability and gait difficulties, cognitive impairment, speaking problems, apathy, impulsivity, and challenges related to the DBS device. (3). EuroQol visual analog scale (EQ-VAS), which records the patient’s self-rated health on a vertical visual analog scale. The endpoints are labeled “The best health you can imagine” and ‘The worst health you can imagine’ rated from 0 to 10 (4). The 8-item version of the Parkinson’s Disease Questionnaire (PDQ-8) 5. The Patient Global Impression Scale (PGI) which is a 7-point self-reported scale that rates the severity (PGI-S) and improvement after treatment (PGI-I).

Complete clinical evaluations through described scales were done at baseline and three months follow-up visits. In between these two assessments, follow-up visits for fine tuning of amplitude intensity and medication were done at week 2, 4, 6, and 9.

### Statistical analyses

Qualitative variables are presented as absolute and relative frequencies, while quantitative variables are presented as medians and their respective interquartile ranges. To detect differences in clinical scales pre- and post-programming, we used paired T-test, Wilcoxon, and McNemar tests according to their distribution. We expected a statistically significant *p* value of <0.05. We conducted our statistical analysis of clinical results using the SPSS package for Mac (version 25 for Mac; IBM, NYC, USA).

### Supplementary information

Related Manuscript File

## Data Availability

All data that support the findings of this study are available upon request to the corresponding author.
