# Supplementary material for: Image-guided programming deep brain stimulation improves clinical outcomes in patients with Parkinson’s disease
Source: NPJ Parkinsons Dis. 2024 Jan 27;10:29. doi: 10.1038/s41531-024-00639-9 (PMC10821897; doi:10.1038/s41531-024-00639-9)
Supplement: Supplementary file 1 — Related Manuscript File [file 41531_2024_639_MOESM1_ESM.pdf]

Corresponding author(s): Dr. Almudena Sánchez-Gómez & Dr. Francesc Valldeoriola

Last updated by author(s): 2023/12/22

Reporting Summary

Nature Portfolio wishes to improve the reproducibility of the work that we publish. This form provides structure for consistency and transparency in reporting. For further information on Nature Portfolio policies, see our Editorial Policies and the Editorial Policy Checklist.

Statistics

For all statistical analyses, confirm that the following items are present in the figure legend, table legend, main text, or Methods section.

n/a	Confirmed	
☐	☒	The exact sample size (*n*) for each experimental group/condition, given as a discrete number and unit of measurement
☐	☒	A statement on whether measurements were taken from distinct samples or whether the same sample was measured repeatedly
☐	☒	The statistical test(s) used AND whether they are one- or two-sided<br>*Only common tests should be described solely by name; describe more complex techniques in the Methods section.*
☐	☒	A description of all covariates tested
☐	☒	A description of any assumptions or corrections, such as tests of normality and adjustment for multiple comparisons
☐	☒	A full description of the statistical parameters including central tendency (e.g. means) or other basic estimates (e.g. regression coefficient) AND variation (e.g. standard deviation) or associated estimates of uncertainty (e.g. confidence intervals)
☐	☒	For null hypothesis testing, the test statistic (e.g. *F*, *t*, *r*) with confidence intervals, effect sizes, degrees of freedom and *P* value noted<br>*Give P values as exact values whenever suitable.*
☒	☐	For Bayesian analysis, information on the choice of priors and Markov chain Monte Carlo settings
☒	☐	For hierarchical and complex designs, identification of the appropriate level for tests and full reporting of outcomes
☒	☐	Estimates of effect sizes (e.g. Cohen's *d*, Pearson's *r*), indicating how they were calculated

Our web collection on statistics for biologists contains articles on many of the points above.

Software and code

Policy information about availability of computer code

Data collection	No commercial, open source or custom code was used to collect data in this study.
Data analysis	Excel was used for analysis and table formulations. Guide XT software was used to image reconstruccions. Statistical analysis of clinical results using the SPSS package for Mac (version 25 for Mac; IBM, NYC, USA).

For manuscripts utilizing custom algorithms or software that are central to the research but not yet described in published literature, software must be made available to editors and reviewers. We strongly encourage code deposition in a community repository (e.g. GitHub). See the Nature Portfolio guidelines for submitting code & software for further information.

Data

Policy information about availability of data

All manuscripts must include a data availability statement. This statement should provide the following information, where applicable:

- Accession codes, unique identifiers, or web links for publicly available datasets
- A description of any restrictions on data availability
- For clinical datasets or third party data, please ensure that the statement adheres to our policy

The data that supports the findings of this study is available from the corresponding author.

Research involving human participants, their data, or biological material

Policy information about studies with human participants or human data. See also policy information about sex, gender (identity/presentation), and sexual orientation and race, ethnicity and racism.

Reporting on sex and gender	The number of male and female patients are described in the text
Reporting on race, ethnicity, or other socially relevant groupings	Race and ethnicitu were not assessed
Population characteristics	Patients with clinical diagnosis of Parkinson´s disease who had undergone bilateral STN-DBS with suboptimal outcome.
Recruitment	Patients from outpatient clinic of the Hospital Clinic of Barcelona with PD who had undergone bilateral STN-DBS
Ethics oversight	The study was conducted following the Declaration of Helsinki and was approved by the local ethics committee at the Hospital Clinic of Barcelona. All patients gave informed written consent before they participated in the study.

Note that full information on the approval of the study protocol must also be provided in the manuscript.

Field-specific reporting

Please select the one below that is the best fit for your research. If you are not sure, read the appropriate sections before making your selection.

☒ Life sciences ☐ Behavioural & social sciences ☐ Ecological, evolutionary & environmental sciences

For a reference copy of the document with all sections, see nature.com/documents/nr-reporting-summary-flat.pdf

Life sciences study design

All studies must disclose on these points even when the disclosure is negative.

Sample size	Our study did not require sample size calculation. All the patient included were directed selected frome the outpatient clinic, based on meeting the inclution and exclution criteria decribed on the text.
Data exclusions	No data was excluded for the analyses
Replication	This is the first study of this kind. All the methods were completly detailed to easly replicate this results.
Randomization	Non randomized.
Blinding	Only the investigator team were blinded on this study. The blinded procedures were conducted by the following individuals: KDG (clinical assessments), VT (programming), AS (GUIDE XT) and FV (follow-up visits). Patients were not blinded to the intervention, they were informed of the possible programming adjustments that would be made, but they were not informed of the specific changes made or lack thereof.

Reporting for specific materials, systems and methods

We require information from authors about some types of materials, experimental systems and methods used in many studies. Here, indicate whether each material, system or method listed is relevant to your study. If you are not sure if a list item applies to your research, read the appropriate section before selecting a response.

Materials & experimental systems

n/a	Involved in the study	
☒	☐	Antibodies
☒	☐	Eukaryotic cell lines
☒	☐	Palaeontology and archaeology
☒	☐	Animals and other organisms
☒	☐	Clinical data
☒	☐	Dual use research of concern
☒	☐	Plants

Methods

n/a	Involved in the study	
☒	☐	ChIP-seq
☒	☐	Flow cytometry
☒	☐	MRI-based neuroimaging

Plants

Seed stocks	NA
Novel plant genotypes	NA
Authentication	NA
